# Consideration of oral health in rare disease expertise centres: a retrospective study on 39 rare diseases using text mining extraction method

**DOI:** 10.1186/s13023-022-02467-7

**Published:** 2022-08-20

**Authors:** Lisa Friedlander, Marc Vincent, Ariane Berdal, Valérie Cormier-Daire, Stanislas Lyonnet, Nicolas Garcelon

**Affiliations:** 1 Université de Paris Laboratoire ECEVE INSERM, UMR1123, Hôpital Robert Debré, Paris, France; 2 Centre de Reference, Maladies Orales et Dentaires Rares, Hôpital Rothschild, APHP, Paris, France; 3 Filière de Santé Maladies Rares TETECOU, Malformations Rares de la tête, du cou et des dents, Hôpital Necker, Paris, France; 4 FHU DDS-Net, Dental School, Université de Paris, Paris, France; 5 Imagine Institute, Data Science Platform, INSERM UMR 1163, Université de Paris, 75015 Paris, France; 6 Service de Médecine Génomique des maladies rares, Hôpital Necker-Enfants Malades, AP-HP, Paris, France

**Keywords:** Rare disease, Network, Oral care, Text mining

## Abstract

**Background:**

Around 8000 rare diseases are currently defined. In the context of individual vulnerability and more specifically the one induced by rare diseases, ensuring oral health is a particularly important issue. The objective of the study is to evaluate the pattern of oral health care course for patients with any rare genetic disease. Description of oral phenotypic signs—which predict a theoretical dental health care course—and effective orientation into an oral healthcare were evaluated.

**Materials and methods:**

We set up a retrospective cohort study to describe the consideration of patient oral health and potential orientation to an oral health care course who have at least been seen once between 1 January 2017 and 1 January 2020 in Necker Enfants Malades Hospital. We recruited patients from this study using the data warehouse, Dr Warehouse® (DrWH), from Necker-Enfants Malades Hospital.

**Results:**

The study sample included 39 rare diseases, 2712 patients, with 54.7% girls and 45.3% boys. In the sample studied, 27.9% of patients had an acquisition delay or a pervasive developmental disorder. Among the patient files studied, oral and dental phenotypic signs were described for 18.40% of the patients, and an orientation in an oral healthcare was made in 15.60% of patients. The overall "network" effect was significantly associated with description of phenotypic signs (corrected *p* = 1.44e−77) and orientation to an oral healthcare (corrected *p* = 23.58e−44). Taking the Defiscience network (rare diseases of cerebral development and intellectual disability) as a reference for the odd ratio analysis, OSCAR, TETECOU, FILNEMUS, FIMARAD, MHEMO networks stand out from the other networks for their significantly higher consideration of oral phenotypic signs and orientation in an oral healthcare.

**Conclusion:**

To our knowledge, no study has explored the management of oral health in so many rare diseases. The expected benefits of this study are, among others, a better understanding, and a better knowledge of the oral care, or at least of the consideration of oral care, in patients with rare diseases. Moreover, with the will to improve the knowledge on genetic diseases, oral heath must have a major place in the deep patient phenotyping. Therefore, interdisciplinary consultations with health professionals from different fields are crucial.

## Background

For the European Union, a disease is considered “rare” if it affects fewer than one in 2000 people. Based on this definition, 27–36 million people affected by a rare disease are estimated in the European Union. Around 8000 rare diseases are currently defined [1, 2].

In France and Europe, territorial university-hospital networks have been created for the management of rare diseases. These Networks are composed of health professionals, researchers, and patient associations. They contribute to the improvement of care, support, training, information, research and innovation. In France, the networking has been set up by groups of pathologies and/or affected systems in the framework of National Plans for Rare Diseases (PNMR). 23 Health Networks for Rare Diseases have been created by the Ministry of Solidarity and Health in agreement with the Ministry of Higher Education, Research and Innovation. They include centres of expertise organized in specialized care by groups of pathologies, covering the whole territory of France. At the European level, French networks are part of ERN Networks; each-one being dedicated to a set of rare diseases that share common aspects.

In the context of individual vulnerability and more specifically the one induced by rare diseases, ensuring oral health is a particularly important issue [3]. Indeed, oral microenvironment is a crossroads of vital functions such as mastication, swallowing, breathing and phonation. Pathogenic oral microbiota induces dental caries, periodontal diseases requiring tooth extractions and treatments with consecutive discomfort, economical costs, and pain. Several oral pathogenic bacteria impact distant organs such as heart or kidney and may worsen several general diseases such as metabolic disorders or ectopic mineralization. Finally, a healthy oral-dental status is part of the person self-esteem and -confidence. Indeed, oral-facial aesthetic handicap, pain or altered facial-buccal expression due to oral diseases impacts the emotional and social life of the individuals. Indeed, the identification of rare diseases with dental and/or oral manifestations [1, 2] has been established disease-by-disease. Individual reports are registered in database of clinical description and/or gene mutation [1, 4] suggesting that 15% rare diseases would affect the oral status and requires dental treatment. This knowledge on oral health in rare diseases is cumulative but not conceived as a global issue.

At the *Necker-Enfants Malades* pediatric hospital (Paris), all the 23 French specific rare disease networks are represented with the presence of more than forty distinct rare disease centres of expertise. All reports of consultations, hospitalizations, complementary examinations, and hospital stays are automatically copied in the Necker Hospital data warehouse (Dr Warehouse®) developed by the Imagine institute. They are processed by using natural language methods to allow physician to handle large sets of data, including narrative reports, particularly for complex rare but also chronic diseases, and to describe the healthcare trajectory of patients. This tool was used here to conduct an unbiased study covering the rare disease networks on oral health and treatment. The objective of the study is to evaluate the pattern of oral health care course for patients with any rare genetic disease. To do so, description of oral phenotypic signs—which predict a theoretical dental health care course—and effective orientation into an oral healthcare were evaluated. This network-by-network analysis will guide efforts to effectively implement dental and oral phenotyping and improve the understanding of genetic diseases.

## Materials and methods

### Study setting and design

We set up a retrospective cohort study to describe the consideration of patients' oral health and their potential orientation to an oral health care course who have at least been seen once between 1 January 2017 and 1 January 2020 in Necker Enfants Malades Hospital.

### Participants

To explore the research question through rare diseases with mostly different clinical signs and being part of distinct rare diseases networks and centres of expertise within the studied hospital, we established a list of representative diseases.

We included patients from 3-year-old with a rare disease (confirmed diagnosis) in the list of selected diseases (Table 1) who consulted between 1.1.2017 and 1.1.2020 at Necker Hospital and whom have been seen at least once in the medical genetics department of Necker Hospital.

**Table 1:** Rare diseases network and diseases selected due the number of included patients

Rare disease	Rare disease French network	Type of diseases
Prader Willi syndrome	AnDDI-Rares	Rare diseases with somatic and cognitive developmental abnormalities
Di George syndrome		
Friedreich ataxia	BRAIN-TEAM	Rare diseases with motor or cognitive expression of the central nervous system
Fallot tetralogy	CARDIOGEN	Hereditary cardiac diseases
Hypertrophic cardiomyopathy		
Dilated cardiomyopathy		
Bourneville tuberous sclerosis	Defiscience	Rare diseases of brain development and intellectual disability
WEST syndrome		
Polymicrogyria		
Rett Sydrome		
X fragile syndrome		
Alagille syndrome	FILFOIE	Rare liver diseases
Sclerosing cholangitis		
Budd Chiari syndrome		
Spinal amyotrophy	FILNEMUS	Rare neuromuscular diseases
Ectodermal dysplasia	FIMARAD	Rare dermatological diseases
Bullous autoimmune dermatoses		
Epidermolysis bullosa		
Neurofibromatosis		
Hirschsprung disease	FIMATHO	Rare abdominal-thoracic diseases
Oesophageal atresia		
Short Bowel Syndrome		
Thyroid ectopia	FIRENDO	Rare endocrine and gynaecological diseases
Turner syndrome		
Cushing's disease		
PFPA syndrome	Fai2r	Rare autoimmune and auto-inflammatory diseases
Congenital hyperinsulinism	G2M	Rare hereditary metabolic diseases
Phenylketonuria		
Willebrand disease	MHEMO	Rare constitutional haemorrhagic diseases
Cystic fibrosis	Muco CFTR	Cystic fibrosis
Alport syndrome	ORKID	Rare renal diseases
Multicystic dysplastic kidney		
Haemolytic uremic syndrome		
Ehlers Danlos syndrome	OSCAR	Rare bone diseases
Osteogenesis imperfecta		
Bardet Biedl syndrome	SENSGENE	Rare sensory diseases
Pierre Robin syndrome	TETECOU	Rare diseases of the head, neck and teeth
Moebius syndrome		
Palate and cleft lip		

The list of rare diseases was determined to perform an analysis based on sufficient data. We firstly considered the three most frequent diseases in each centre of expertise of Necker Hospital. To do so, we screened this list including 123 rare diseases into the National Data Bank on Rare Diseases, which collates all the data filled in by the centres of expertise. To select the conditions to be included in the study, we grossly reviewed all medical records of patients meeting the inclusion criteria for all conditions. Conditions for which the data in the medical records were poorly populated were excluded, ending with 39 identified rare diseases. We recruited patients from this study using the data warehouse, Dr Warehouse® (DrWH), from Necker-Enfants Malades Hospital. DrWH is a document-based open-source data warehouse oriented toward narrative clinical reports from the Electronic Health Records (EHRs). It contains more than 7.5 million clinical free-text documents produced at Necker Hospital from 2009, for more than 700,000 individuals and more than 20 medical departments. It allows searching for patients from structured data (e.g. biology) and free text (e.g. hospital reports) [5–7]. Dr Warehouse is a hospital data warehouse automatically fed by copying the electronic health records and other sources of data produced during the care of the patients. We used Dr Warehouse search engine to extract features localized in the free text (narrative reports) and almost never in the coded data (ICD10). All our questions and hypotheses are based on the way in which doctors fill in patients' medical records in their own words and not through specific coding.

The study population was constructed on each pathology studied using the Dr Warehouse® search engine by entering the inclusion criteria and the name of the concerned pathology. The sample selection was done in Dr Warehouse by a keyword search: “disease name” was used. Each patient was manually reviewed to exclude patients with an unconfirmed diagnosis.

### Data collection

Dr Warehouse provides the functionality (called eCRF) to set up a clinical search form directly in the user interface. This tool was designed to facilitate the extraction of variables of interest from the EHR. For each variable, it proposes a value extracted from the EHR and displays the text area containing the items sought in the reports. The user must validate or modify the automatically extracted values.

We defined the variables (1) semi automatically extracted from textual data: main diagnosis (based on the list of rare diseases), oral status, phenotypic signs description (yes or no), orientation in an oral health care course, (2) automatically extracted from structured data: gender, age, postal code of residence, number of consultations in the hospital, number of hospitalizations in the hospital, number of different medical departments consulted, (3) and manually filled: Fdep (French DEPrivation index), index of social deprivation according to the postal code of residence), presence of a pervasive developmental disorder, and inclusion in a rare disease network.

To determine oral status, we searched for French words beginning with "dent" to find all clinical reports containing words in the field of dentistry (i.e., equivalent in English: tooth, teeth, dental, dentist, dentition etc.). We added in the research the following words to extend the semantic field: “carie (decay), gencive (gum), oral (oral), bouche (mouth). With this method, we were able to retrieve all patients with records mentioning these keywords.

### Statistical analysis

#### Descriptive statistics

A descriptive analysis of the database generated was carried out. Categorical variables were summarized by frequency and percentage. Continuous variables were summarized by mean (1st; 3rd quartile), minimum and maximum.

Total patient data cross-tabulated with Social Deprivation Factor data, and information about the pathways caring for the patients. The FDEP was cross-referenced to the original table via zip code and a zip code to commune code conversion table. The original match data did not include a Paris commune code. They were replaced by the INSEE 2018 census data. The FDEP (2008) per postal code was calculated by averaging the FDEP of the corresponding communes weighted by their respective populations (2018).

The care course data were cross-referenced to the patient data via the actual principal diagnosis (i.e., the first value in the 'Principal diagnosis' column that contains more than one).

#### Analytical statistics

The model applied was a logistic regression where the output (dependent) variable was either the orientation in an oral healthcare or the description of oral phenotypic signs.

The importance of each predictor variable of interest was assessed using a likelihood ratio test comparing a full model containing said variable to a restricted model void of it. When deemed necessary, additional covariates were added to both models to control for potential confounding effects.

We were mainly interested in the effect of the services visited on oral health care. We started by testing the variables that will be used to fit the variables describing the services with models including only these variables.

Then we tested the variables describing the services visited globally (i.e., 1 variable = all the services visited) by including in the comparison model the variables to be adjusted. All the statistical tests were bilateral with a significance level of 5%, which means that a *p* value < 5% was considered significant.

All the variables tested up to this stage were corrected by the Bonferroni procedure applied to the number of variables tested * nb of outcomes. In total, we had 2 outcomes (phenotypic signs/orientation, 8 variables tested: Age, Sex, number of hospitalizations, number of consultations, number of different medical services, oral status, FDEP).

## Results

### Descriptive data

The sample studied included 2712 patients, children, and adults with 39 different pathologies, in 17 different rare disease networks.

The rare diseases studied and the networks they belong to were described in Table 1. We made our extraction on 39 rare diseases representing, almost in an exhaustive way, all the rare disease networks created in France and present at the Necker-Enfants-Malades hospital.

The study sample included 2712 patients, with 54.7% girls and 45.3% boys (Table 2). The population was adult and paediatric with an average age of 19.1 years and a median age of 14.5 years (Table 3). In the sample studied, 27.9% of patients had an acquisition delay or a pervasive developmental disorder. Among the patient files studied, oral and dental phenotypic signs were described for 18.40% of the patients, and an orientation in an oral healthcare was made in 15.60% of patients (Table 2).

**Table 2:** Description of the study population: categorical variables

Variables		n = 2712	%
Sex	Female	1494	55.1
	Male	1218	44.9
Age repartition	< 18 YO	1577	58.1
	≥ 18 YO	1135	41.9
Description of phenotypic signs	Yes	500	18.4
	No	2212	81.6
Orientation in an oral health care	Yes	422	15.6
	No	2290	84.4
Oral condition	Very bad	77	2.8
	Bad	189	7.0
	Good	211	7.8
	Not filled	2235	82.4
Presence of a pervasive developmental disorder	Yes	758	27.9
	No	1954	72.1

**Table 3:** Description of the study population: continuous variables

Variables	n	Mean	Std	25%	50%	75%	Min;max
Number of hospitalizations	2712	2.24	4.25	0	1	3	0;51
Number of consultations	2712	26.7	39.5	6	15	31	0;752
Median length of stay (days)	2712	3.38	6.85	0	1.54	4.13	0;101
Number of different medical services attended	2712	5.14	3.62	2	4	7	0;23
Fdep	2712	− 0.73	1.28	− 1.81	− 0.557	0.305	− 4.22;2.35
Age	2712	19.1	14.5	9	15	23	3;93

We also looked, adjusted on age, at the average number of consultations per patient (26.7), the average number of hospitalizations (2.24), the median length of hospital stays in days (3.38), and the average number of different hospital medical services that patients attended (5.14). We also calculated the average FDEP (− 0.73) (Table 3).

**Fig. 1:**
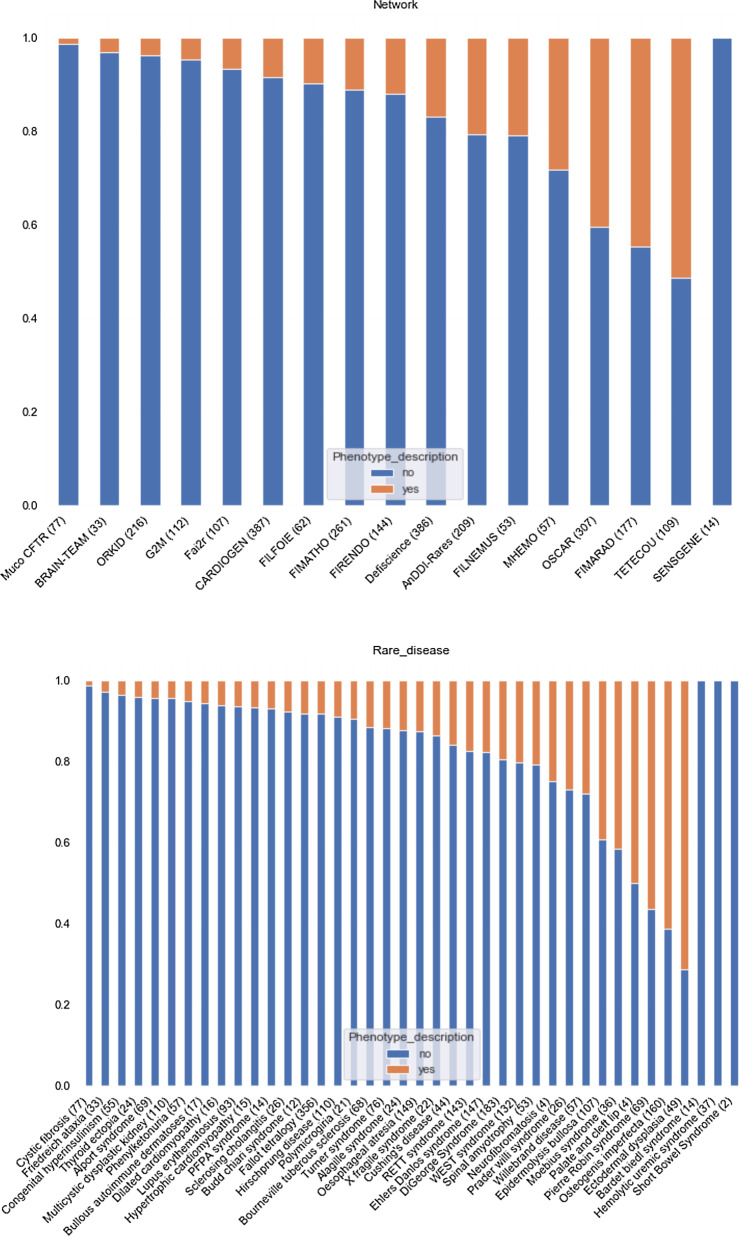
Description of phenotypic signs according to the patients' rare disease network and disease. Counts were ordered by proportion of phenotype description. Count of patients are indicated in parentheses

**Fig. 2:**
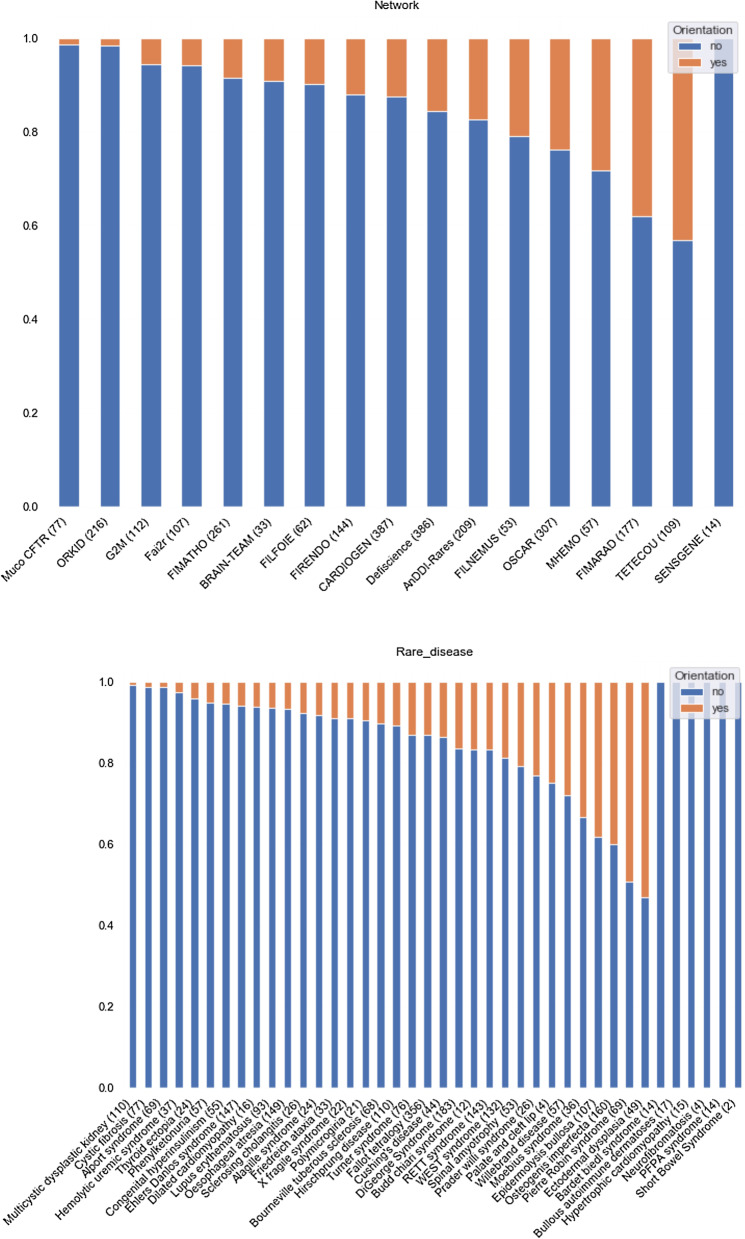
Description of orientation in an oral healthcare according to the patients' rare disease network and disease. Counts were ordered by proportion of orientation. Count of patients are indicated in parentheses

We studied the distribution of our variables of interest (description of phenotypic signs (Fig. 1) and orientation in an oral healthcare (Fig. 2) according to the patients' rare disease network and disease.

Networks whose practitioners most frequently noted oral phenotypic signs and referred the most patients for oral care were the TETECOU (rare diseases of the head, neck, and teeth), FIMARAD (rare dermatological diseases), OSCAR (rare bone diseases), and the MHEMO (haemophilia and other constitutional bleeding diseases) networks. In terms of pathologies, the oral phenotypic signs were most noted in patients with ectodermal dysplasia, osteogenesis imperfecta, and Pierre Robin syndrome (Fig. 1).

### Analytic data

About orientation in an oral care course, we show a significant statistical association about the number of hospitalizations (corrected *p* = 2.81e−10), the number of consultations (corrected *p* = 7.16e−122), and the number of medical services attended (corrected *p* = 5.35e−22).

Regarding the description of oral phenotypic signs, we show a significant statistical association with respect to the number of hospitalizations (corrected *p* = 9.74e−07), the number of consultations (corrected *p* = 1.71e−13), and the number of medical services attended (corrected *p* = 8.15e−25), and the FDEP index (corrected *p* = 0.02) (Table 4).

**Table 4:** Association between the variables studied and orientation in an oral healthcare/description of oral phenotypic signs

Variables		Orientation*				Description of phenotypic signs**			
		*p* value	*p* value cor	OR	95% IC	*p* value	*p* value cor	OR	95% IC
Age		0.11*	1	0.99	0.99;1.00	0.02*	0.29	0.99	0.98;1
Gender [ref = F]	M	0.26	1	1.13	0.88;1.33	0.10*	1	1.18	0.95;1.39
Number of hospitalizations (age)		1.75e−11*	**2.81e−10**	1.07	1.05;1.09	6.09e−08*	**9.74e−07**	1.06	1.03;1.08
Number of consultations (age)		4.47e−13*	**7.16e−12**	1.01	1.006;1.01	1.07e−14*	**1.71e−13**	1.01	1.007;1.01
Number of different medical services attended (age)		3.35e−23*	**5.35e−22**	1.14	1.12;1.18	4.06e−25*	**6.5e−24**	1.14	1.12;1.17
Fdep		0.00264*	0.04	1.13	1.04;1.23	0.00*	**0.02**	1.14	1.05;1.22
Presence of a pervasive developmental disorder [ref = no]	yes	0.799	1	1.03	0.84;1.35	0.23	1	0.87	0.73;1.13

Bolded numbers represent significant values

When covariates adjustment is included the names of the covariates are indicated within parentheses. When the variable is categorical the reference value used is indicated in square brackets. <*p*> is the uncorrected *p* value, <*p* value cor> the corrected value (using a Bonferroni procedure with n test = 16), a *p* value with an asterisk * indicates inclusion as a covariate in further models based on a threshold of 0.2 on the nominal uncorrected *p* value

*Covariates selected for the outcome “Description of phenotypic signs”, are number of consultations from beginning, number of hospitalizations from beginning, FDEP, number of different medical services attended, age, gender

**Covariates selected for the outcome “orientation” are: number of consultations from beginning, number of hospitalizations from beginning, FDEP, number of different medical services attended, age

We wanted to know if the rare disease network in which the patients whose data were studied were statistically associated with the description of phenotypic signs and orientation to oral care course. To do this, we studied the overall "network" effect (Table 5).

**Table 5:** Global network effect

Network	Phenotypic description				Orientation				
	*p*	p*	OR	95% CI	*p*	p*	OR	95% CI	Nobs full
AnDDI-Rares: rare diseases with somatic and cognitive developmental abnormalities	1.8e−78	1.44e−77	**1.17**	0.74;1.84	4.47e−45	3.58e−44	1.02	0.63;1.64	2711
BRAIN-TEAM: rare diseases with motor or cognitive expression of the central nervous system	1.8e−78	1.44e−77	0.19	0.02;1.38	4.47e−45	3.58e−44	0.68	0.19;2.35	2711
CARDIOGEN: hereditary cardiac diseases	1.8e−78	1.44e−77	0.51	0.32;0.82	4.47e−45	3.58e−44	0.88	0.57;1.34	2711
FILFOIE: rare liver disease	1.8e−78	1.44e−77	0.51	0.19;1.31	4.47e−45	3.58e−44	0.55	0.22;1.40	2711
FILNEMUS: rare neuromuscular diseases	1.8e−78	1.44e−77	**1.43**	0.67;3.01	4.47e−45	3.58e−44	**1.53**	0.72;3.21	2711
FIMARAD: rare dermatological diseases	1.8e−78	1.44e−77	**4.49**	3.18;7.52	4.47e−45	3.58e−44	**3.88**	2.51;5.99	2711
FIMATHO: rare abdomin-thoracic diseases	1.8e−78	1.44e−77	0.52	0.31;0.85	4.47e−45	3.58e−44	0.41	0.24;0.71	2711
FIRENDO: rare endocrine and gynaecological diseases	1.8e−78	1.44e−77	0.72	0.38;1.34	4.47e−45	3.58e−44	0.75	0.40;1.39	2711
Fai2r: rare autoimmune and auto-inflammatory diseases	1.8e−78	1.44e−77	0.30	0.13;0.72	4.47e−45	3.58e−44	0.25	0.10;0.63	2711
G2M: rare hereditary metabolic diseases	1.8e−78	1.44e−77	0.24	0.09;0.62	4.47e−45	3.58e−44	0.27	0.10;0.66	2711
MHEMO: rare constitutional hemorrhagic diseases	1.8e−78	1.44e−77	**1.96**	0.98;3.88	4.47e−45	3.58e−44	**2.01**	1.01;3.98	2711
Muco CFTR: cystic fibrosis	1.8e−78	1.44e−77	0.01	0.00;0.09	4.47e−45	3.58e−44	0.01	0.00;0.13	2711
ORKID: rare renal diseases	1.8e−78	1.44e−77	0.23	0.10;0.50	4.47e−45	3.58e−44	0.09	0.02;0.28	2711
OSCAR: rare bone, calcium and cartilage diseases	1.8e−78	1.44e−77	**5.23**	3.56;7.67	4.47e−45	3.58e−44	**2.2**	1.47;3.30	2711
SENSGENE: rare sensory diseases	1.8e−78	1.44e−77	9.92e−10	0;inf	4.47e−45	3.58e−44	1.1e−09	0;inf	2711
TETECOU: rare diseases of the head, neck and teeth	1.8e−78	1.44e−77	**3.97**	2.44;6.44	4.47e−45	3.58e−44	**3.30**	2.02;5.37	2711

Bolded numbers represent significant values

*Adjusted on covariables: number of consultations from beginning, number of hospitalizations from beginning, FDEP, number of different medical services attended, age, gender

The “overall network effect” was significantly associated with description of phenotypic signs (corrected *p* = 1.44e−77) and orientation to an oral healthcare (corrected *p* = 3.58e−44).

Taking the Defiscience network (rare diseases of cerebral development and intellectual disability) as a reference for the Odd Ratio analysis, OSCAR, TETECOU, FILNEMUS, FIMARAD, MHEMO networks stand out from the other networks for their significantly higher consideration of oral phenotypic signs and orientation in an oral healthcare than the other networks. Concerning the Anddi-Rares network, a slight effect is significantly shown regarding the description of phenotypic signs (0R = 1.12) but not regarding the orientation in the oral care pathway.

## Discussion

### Discussion of the results

This study gives access to a macroscopic picture on rare diseases in a representative population. Firstly, the results showed that the network effect is strong. Not every rare disease network had the same way of considering oral health. The networks that stand out in their consideration of oral health were those whose diseases have, in an almost exhaustive way, recognized constitutional oral or dental phenotypic signs. Consistently, a first group of rare diseases/networks emerged which was associated with dental and oral health in the literature [1, 4].

The French Head, Neck and Teeth TETECOU was by its nature the first identified network. Other evidenced networks were the ones for which a dental defect may be the first call sign of syndromes they take in charge [8, 9], such as for instance: significant tooth agenesis or oligodontia in ectodermal dysplasia (FIMARAD), enamel defects, tooth eruption impairment and gingival fibromatosis in enamel renal syndrome [8] (OSCAR), keratocystic odontogenic tumors (KCOT) in Gorlin syndrome [9], or unique upper central incisor in holoprosencephaly (AnDDi-Rares). These networks entertain numerous exchanges with the centres of expertise for rare oral and dental manifestations (TETECOU). The FIMARAD, which deals with rare dermatological pathologies such as ectodermal dysplasia, hosts a number constitutional dental and oral phenotypes as various degrees of oligodontia with other dental abnormalities [10]. The results of the global analysis of the network effect and the analysis of the odd ratios allowed us to distinguish each of the rare disease networks. Certain networks, such as the OSCAR, had, in any case in the information that we were able to collect, a fairly broad consideration of oral health due to the specificity of the pathologies taken in charge in its networks (i.e. Ehlers Danlos Syndrome, Osteogenesis imperfecta). Indeed, in our data, these networks stand out from the others in their great consideration. This appeared in the extraction of textual data, in the oral phenotypes and proposal of orientation for oral care.

For patients with rare disease outside these 3 networks, the dental and oral phenotype may be either insufficiently taken in charge or even unknown, and thus care may not be referred to existing expert sites. In addition, despite the efficient territorial network of oral health services set up in the PNMR1-3 national plans, a significant number of patients do not seek oral care [11]. There were many reasons for this as the complexity of their pathology and/or their disability requiring experts and/or hospital structures that were difficult to access in the territories and, in some cases, the absence of dental and oral professionals in rare disease centres leading their medical care course. Finally, even when the structures and experts are reached, some financial issues may be raised [12].

In contrast, most other rare diseases are not genetically related to dental symptoms. For some of them it is expected that the oral status is deficient due to indirect relationships with functions and pathogenic microbiota. This is the case for instance in motor or neurological handicaps which alter patient capacities in controlling body hygiene, such as the TETECOU and AnDDi-Rares but also the previously not identified FILNEMUS (rare neuromuscular diseases) highlighted here. AnDDi-Rares, specialized in the care of rare diseases with somatic and cognitive developmental abnormalities, takes care of patients presenting in most of the cases of delay of acquisitions and disorders of cognitive development. Affected patient handicaps initiate and entertain the pathogenic cascade due to uncontrolled oral biofilms: decay, periodontal disease, tooth extraction, pain, and distant secondary targets for the bacteria. This ends with eating disorders, in the worse situations’ cachexia, for these handicap and finally edentulous patients. This is easily explained by the multiplication of hospital stays, the multiplication of practitioners met in hospital, with different knowledge and sensitivity to oral medicine. One hypothesis that may explain this is that patients treated in the AnDDi-Rares and TETECOU have severe craniofacial features and need paediatric surgery very early [13, 14]. Indeed, access to a dental care adapted to their rare pathology often appears to be deficient [1, 15]. The results showed significantly that the higher the number of hospitalizations, the higher the number of consultations and the higher the number of different medical services attended by the patients, the more oral phenotypic signs are described by the practitioners and the more there is an orientation of the patients in an oral healthcare. To suffer from a pervasive disorder of the development is not significantly associated with a potential orientation in a course of oral care nor with the description of the oral phenotypic signs. These results, from the univariate analysis, differ slightly from the multivariate analysis with the analysis of the overall effect of networks on the variables of interest.

MHEMO (constitutional haemorrhagic pathologies, here Willebrand disease) also shows an important consideration of oral health, in any case significantly more important than the other networks explored. This could be explained by the nature of the pathologies treated, in this case Willebrand disease, a pathology with important hemorrhagic risks and which must have an adapted oral management [16, 17].

Five networks we studied fill in the patient database while their phenotypes predict that dental and oral phenotypes may raise difficulties. The analysis of the collected data suggests a lack of connection with oral professionals during early childhood and adult transition. These networks are BRAIN-TEAM with Friedreich ataxia (Rare diseases with motor or cognitive expression of the central nervous system), G2M with Congenital hyperinsulinism and Phenylketonuria (Rare Hereditary Metabolic Diseases), Muco CFTR (Cystic Fibrosis), ORKID with Alport syndrome, Multicystic dysplastic kidney, Haemolytic uremic syndrome (Rare Renal Diseases), and SENSGENE with Bardet Biedl syndrome (Rare sensory diseases). Interestingly, some of these diseases have oral phenotypic signs already known, as Bardet-Biedl syndrome, a monogenic autosomal recessive nonmotile ciliopathy, as an archetypal condition and dental anomalies are present in a majority of individuals affected due to abnormal embryonic orofacial and tooth development [18].

Other diseases have effects on oral health because of the patients’ treatments. For example, patients with phenylketonuria need to follow a very specific diet. Diet plays an integral role in the maintenance of oral health, dietary modifications due to medical problems such as phenylketonuria can have adverse effects on oral health [19]. For Alport syndrome, a study showed the significance of gingival biopsy in the initial diagnosis and periodontal evaluation after renal transplantation and changes in the gingival tissues with patients under therapy with cyclosporin-A [20]. It has been shown for Cystic Fibrosis, that these patients have a high caries risk and that early management is necessary [21].

It clearly appears that an early consideration of the oral phenotypes of the pathologies and an early orientation of the patients in an adapted oral management would make it possible to improve the knowledge on these diseases and to improve the oral quality of life of the patients.

### Critical analysis of the study

Dr Warehouse search engine was used here to select a population based on oral features and perform an exhaustive analysis in a rather large population (800,000 patients and 8 million health reports). Necker Hospital is strongly involved in the national organization, constituted by the centres of reference for rare diseases and by the rare disease health networks. In this hospital, there are 59 expertise centres on rare diseases, representing 23 rare disease health networks. This offers an exceptional prevalence of patients with rare diseases. We have discarded many diseases that were really very rare and for which the number of patients would not have been sufficient to have a fine analysis of the contents of the medical files. The most representative diseases were selected. Those where we could get the maximum number of patients in the shortest time. With this method we were able to select 2712 patients, which we considered as acceptable sample for the analysis.

The semi-automatically generated eCRF allows avoiding missing data in an almost exhaustive way. We were able to perform the analysis on 2711 patients. Using this method, inclusions could be made in a very short period for the size of the sample (less than 3 months, about 50 h of work).

To carry out the cohort, depending on the pathologies, the words "dent*, caries, gum, oral, mouth" were used. These choices may be debatable, but a trade-off was necessary to make the analysis. Although we have shown that these words were the best choice for the broadest possible data extraction, practitioners may talk to patients about their oral health without necessarily writing it down in the records. Indeed, if patients report to their physicians that oral health care is effective, and performed close to home for example, this may not appear in the records, yet it was discussed during the consultations. This shows the limitation of analysing structured and unstructured data from patient records completed by physicians. If the information is not written in the records, it cannot be extracted and thus analysed. We systematically searched through the patients’ health reports for the most common terms used by physicians to describe oral health. Surprisingly, we came to the conclusion that the 3 main terms were "good”, “bad” and “very bad". They may seem vague and imprecise, but they are actually the terms used to describe oral health. This lack of precision was an important point that we wanted to raise in this work. This description of oral status is very subjective to the one who writes them. So it depends on the accuracy, and the expertise of the practitioner who fills in the file. This is the whole point of the analysis we have carried out in this work. By deciding to extract unstructured data, i.e. not coded, according to a particular classification, we wanted to show, that the doctors who fill in the medical data use a rather poor vocabulary when it comes to describing oral health. These doctors are eminent specialists in their pathology, yet they use a very simple vocabulary when it comes to describing oral health. One of the aims of this study is to demonstrate this point.

Regarding the main evaluation criteria, the description of phenotypic signs and the orientation in an oral health care pathway, the proportions of patients are quite low, less than 20% for these two variables. The presence of a pervasive developmental disorder was studied because it can be an obstacle to an oral examination in good conditions as well as a limit in the access to dental care for patients.

Considering the ecological indicators, we used the French DEPrivation index (FDep), which was developed specifically for the French context and has been used in the past to study the impact of deprivation on the use or consumption of care [22, 23]. The higher the score, the greater the social disadvantage. We wanted to know if the fact of living, in a more disadvantaged commune could have a link with a less good oral health which would have been considered in a more significant way by the doctors in charge of the pathology of patients. We found a significant association between the final description and the Fdep and therefore a more disadvantaged population, in patients who have more oral phenotypic signs. The French median FDEP is 2, ranging from − 6 to 10. The average FDEP of the patients in our study is − 0.72, that is located in the first quintile of the National FDEP, situating our population as rather privileged compared to the French population [24]. This situation may come from the geographical location of Necker hospital in the Ile de France.

We clearly see that in the rare disease networks where the diseases treated have an imoortant oral component, the management, or at least the consideration of oral health, is more emphasized than in the other networks. Indeed, oral health is obviously based on hygiene, maintenance and nutrition, but also on the severity of the rare disease, place of oral health among the patient's other pathologies and their severity, presence of a pervasive developmental disorder and neurodevelopmental delay, and on socio-economic parameters. One of the innovative elements of our work is that until now the way in which the oral health of patients with rare diseases was assessed had never been performed by the way in which doctor’s spoke in medical records. This study highlights the points to be worked on in collaboration with the teams that take care of these rare disease patients to significantly advance the consideration of oral health.

## Conclusion

The expected benefits of this study are, among others, a better understanding, and a better knowledge of the oral care, or at least of the consideration in patients affected by rare diseases. There is a strong case for better promotion of oral health in centres of expertise, for diseases that are not intuitively linked to oral health. This promotion will involve prevention, therapeutic education, and better awareness of health professionals on oral health. These measures will improve the oral health care pathway for patients with rare diseases.

Moreover, with the will to improve the knowledge on genetic diseases, oral heath must have a major place in exhaustive patient’s phenotyping. Therefore, interdisciplinary consultations with health professionals from different fields are crucial. The presence of an odontologist, carrying out a precise oral phenotyping, as it already exists in some rare disease expertise centres in France and Europe, will bring a major and primordial lighting on the knowledge of rare diseases. It will also allow us to orient patients in an adapted oral care pathway.

## Data Availability

The datasets during and/or analysed during the current study are available from the corresponding author on reasonable request.
